# General Therapeutics and Materia Medica

**Published:** 1845-01

**Authors:** 


### GENERAL THERAPEUTICS AND MATERIA MEDICA. By *Robley Dunglison*, M.D. Professor of the Institutes of Medicine, &c. Philadelphia, 2 vols. 8vo. 1843.

IT is gratifying to all those who were personally acquainted with Dr. Dunglison in Modern Babylon, to find that he has distinguished himself in the far-West, and carried to his new and adopted country, talents and acquirements that would have insured him success and reputation even in this over-stocked land of literature, science, and art. Emigration is not confined to the labourers in the field, mechanics in the work-shops, or artists in their atteliers. The paths to fame and fortune, in these isles, are so densely crowded—so long and tortuous—so steep and rugged—that many of the climbers either faint by the way, or despond and give up the attempt—while few, comparatively, reach the goal of ambition, wearied and damaged by the toilsome ascent. No wonder, then, that some of our brethren cross the Atlantic, in search of a wider theatre, a freer air, and a less densely crowded field for the exertion of their talents and industry.

The work before us is divided into two Parts—THERAPEUTICS and MATERIA MEDICA. The author modestly designates it as "a text-book" for students—but we are all students, during the whole of our career, and not a few of us require a fillip to our memories—a new wrinkle in our horns—and the frequent rehearsal of old lessons, from time to time. Still the volumes are essentially elementary, and consequently adapted particularly for the student—though the full-grown practitioner may often glean from them new facts as well as original ideas. As an elementary work, it is insusceptible of analysis, and we can only introduce to our readers a few specimens of the style and matter of which it is composed, in order that they may form some judgment of its execution. Speaking of Prejudices, Dr. Dunglison remarks that :—

"A prejudice is still found, however, against the use of iced water in fever where calomel is given. The feeling exists strongly in many parts of the Southern and Middle States, but it is rapidly yielding, and ought to be altogether abolished. Some cases have occurred in which individuals have caught cold, or have had disagreeable symptoms supervening, after cold water has followed calomel; but they have been cases of the *post hoc,* rather than of the *propter hoc.* The author has been for years in the habit of allowing the use of iced water after calomel in fevers, and has never had the slightest evidence of any disagreeable results from it." 58.

We have never hesitated to apply ice to the head, when the brain is affected, whatever calomel may be introduced into the stomach; and we have rarely found that cold drink produced griping or other inconvenience, during the use of mercury in fevers.

The doctrine of Homœopathy does not appear to captivate the senses of our author, no more than Mesmerism.

"One of the strangest of the assertions of Hahnemann and his followers is—that homœopathic medicines acquire at each division or dilution a new degree of power, by the rubbing or shaking to which they are subjected, 'a means,' says Hahnemann, 'of developing the inherent virtues of homœopathic medicines that was unknown till my time; and which is so energetic, *that latterly I have been forced, by experience, to reduce the number of shakes to two, of which I formerly prescribed ten to each dilution.*'" 82.

What tremendous powers might thus be called forth by *agitation!* Daniel O'Connell has evidently caught the idea from Hahnemann, and has determined to shake the Repeal question till it overpowers and lays the Saxon in the dust!

Speaking of the therapeutical agency of Nauseants, Dr. D. remarks :—

"In *constipation,* a union of nauseants with cathartics becomes occasionally adviseable, and at times effectual, after cathartics alone have been unsuccessfully employed. If the constipation be dependent upon any irritated condition of the exhalents of the canal, the use of debilitants,—such as those now under consideration,—reduces the erethism, and facilitates the operation of the purgative. Whenever, too, it is desirous to break in upon a morbid chain, and especially in the neuroses, nauseants may be beneficially administered: but, in these cases, the revulsion, induced by a nauseating emetic, is generally preferred, in consequence of the more powerful impression which it makes on the nervous system." 92.

Small proportions of tartrate of antimony, combined with colocynth and the pil. hydrarg., we have been long in the habit of using in the obstinate constipation of bowels that too often accompanies dyspepsia and torpidity of liver.

### *Enemata.*

That Dr. Dunglison and nine-tenths of the profession labour under erroneous notions as to the physiological, medicinal, and mechanical effects of enemata, we have had ample opportunities of knowing.

"When thrown in contact with the lining membrane of the *rectum* they irritate it; and, by sympathy of continuity, their influence is extended to the upper portion of the tube. Hence, they may be administered with advantage, when cathartics cannot be easily given by the mouth, as where deglutition is impracticable." 139.

Now we know, from twenty-five years of personal, as well as general experience in regard to enemata, that it is not merely by *sympathy*, but by general distention of the colon—sigmoid flexure and transverse arch—that the effects of enemata are produced. Of what use would be a few ounces of fluid, to distend the rectum? None at all. Besides the rectum is not really distended till near the close of the operation. The fluid ascends into the transverse arch of the colon much more easily than it distends the strong muscular tube of the rectum. Any one who is accustomed to use the syringe, will feel the fluid, after two or three strokes of the piston, run along the transverse arch, and when that portion of the bowel is filled or excited into contraction, the rectum is distended, and the enema can no longer be retained. The best mode of using a lavement is to put two or three pints of warm water into a basin—wash the hands with Windsor or other soap—and then pump the soap-suds gently and slowly up the gut, till re-action takes place, and the enema and fæces are discharged. It is all very well for the French lady, or the English invalid, to throw up a pint or so of warm water, and then lie down for an hour or two to knead the bowels and push the fluid about in the colon. But the lawyer, the merchant, the doctor, or the man of business, cannot go through this tedious process, and must use the summary one which we have described—and which, after all, is the "cito, tute, ac jucundo" operation.

We can only spare space for one more quotation, and that is from the second volume, under the head of "BLOOD-LETTING," upon which important therapeutical agent Dr. Dunglison has made many judicious and important remarks.

"It may be laid down, perhaps, as a general law, that when blood is lost to a considerable amount the great nervous centres receiving an inadequate—and the rest of the nervous system an irregular—supply, their excitability becomes largely and irregularly developed, so that, under this impressible condition of the nerves, the blood-vessels, whose functions are carried on under their presidency, assume augmented action; and if, owing to the previous existence of hyperæmia in any organ, the nerves, proceeding to that organ, are in a morbidly excitable condition, a fresh development of excitability may ensue after the bleeding, and the hyperæmic condition, instead of being relieved by the loss of blood, may be augmented by it. In individuals, whose nervous system is very impressible, the same effects may be induced by a full bleeding, as have been described to result from excessive discharges from the uterus, and, accordingly, where hyperæmic conditions occur in such individuals, the practitioner is cautious in the use of the lancet, and if he employs it, he does not carry the depletion so far as to depress too much the powers of the system,—aware, that if he should do so, under the nervous irritability or neuropathia, which he develops, re-action might succeed to such an extent as to reproduce the exaltation of organic actions in the part, and perhaps to a greater degree than before the operation. It is in such impressible habits that advantage is found in the adoption of other sedative agents; and that a combination of blood-letting, short of inducing syncope, with a full sedative dose of opium, is often so serviceable;—the bleeding diminishing the exaltation of the vital manifestations, by acting on the nerves through the bloodvessels; and the opium preventing the subsequent development of nervous excitability. In strong individuals, the same plan, pushed to a still greater extent, is equally successful and not the less philosophical, when employed for the removal of internal inflammations. It is the plan which, as before observed, is adopted with so much success, in acute peritonitis;—the bleeding being carried so far as to make a decided impression on the system, and the opium administered in a full dose; a sedative influence is thus exerted on the body generally, and on the inflamed tissue in particular, under which the hyperæmia is effectually subdued." 151.

Our junior brethren in America will find in these volumes of Dr. Dunglison, a "Thesaurus Medicaminum," more valuable than a large purse of gold.

